# Phenytoin and Rifampin Do Not Decrease Levels in Acute Tacrolimus Toxicity

**DOI:** 10.1177/2324709618765862

**Published:** 2018-03-24

**Authors:** Benjamin O. Lawson, Heemesh Seth, Dan Quan

**Affiliations:** 1 Honor Health, Internal Medicine Residency, Scottsdale, AZ, USA; 2 Maricopa Integrated Health System, Department of Emergency Medicine, Phoenix, AZ, USA; 3 Department of Emergency Medicine, College of Medicine, University of Arizona, Phoenix, AZ, USA

**Keywords:** tacrolimus toxicity, rifampin, phenytoin, bone marrow transplant

## Abstract

Tacrolimus is used in bone marrow transplant patients to prevent graft-versus-host disease. There have been few case reports of tacrolimus toxicity (>30 ng/mL) in solid organ recipients as well as in nontransplant patients. Several case reports suggest phenytoin and rifampin decrease tacrolimus levels in toxicity, but does it actually make a difference? A 60-year-old man with acute myeloblastic leukemia after allogenic stem cell transplant with fever, diarrhea, and abdominal pain was transferred to the intensive care unit for persistent hypotension and acute hypoxic respiratory failure requiring intubation. The following day his tacrolimus level was 8.6 ng/mL and creatinine was 2.2 (baseline = 1.8). The patient inadvertently received 15 mg intravenous tacrolimus instead of his scheduled 0.5 mg intravenous. Four hours later, a random tacrolimus level was 36.4 ng/mL. Tacrolimus was discontinued; phenytoin 200 mg BID was started for 4 doses and rifampin was started for 2 doses at 600 mg. Sixteen hours postinjection, tacrolimus level decreased to 26.4 ng/mL and to 9 ng/mL after 64 hours. Creatinine improved to 1.1 after 30 hours. He was extubated 5 days later without any new neurological findings and his creatinine returned to baseline. Our patient received 30 times his daily dose resulting high tacrolimus levels. Assuming there was sufficient time for distribution, our patient’s half-life increased to 34.5 hours compared with the reported half-life of 12 hours. The possibilities for this increase include ineffective or harmful effects of the phenytoin/rifampin combination, change in metabolism kinetics at high levels, or other unidentified patient-specific factors. Further studies should be done to ensure that phenytoin and rifampin are safe to give in tacrolimus toxicity.

## Introduction

Tacrolimus was discovered in 1984 from a soil sample at the base of Mount Tsukuba in Tokyo.^1^ In its early times, it was referred to as FR000506 and found to suppress interleukin-2 production.^1^ Suppressing interlukin-2 results in preventing T-cell activation, differentiation, and proliferation.^1^ Tacrolimus is mostly metabolized by the P450 cytochrome (CYP) 3A4 isoenzymes in the liver^2^ and P-glycoprotein located among the intestinal mucosa.^3^ Nephrotoxicity, neurotoxicity, infections, diarrhea, and disturbances in glucose metabolism have all been linked to tacrolimus.^4^

The first allogeneic hematopoietic stem cell transplantation (HSCT) was performed by E. Donnall Thomas in 1956.^5^ The major complication of allogeneic HSCT is graft-versus-host disease, and this complication can be prevented by inhibiting calcineurin, which is necessary for activating T cells.^6,7^ Tacrolimus is used to prevent activation of T cells, thereby preventing graft-versus-host-disease to occur in HSCT. Tacrolimus concentration has to be tightly regulated and at times can reach toxic levels. There have been a few case reports of tacrolimus toxicity (>30 ng/mL) in solid organ recipients^8^ as well as in nontransplant patients.^9^ Several case reports suggest phenytoin and rifampin as a potential treatment option to decrease tacrolimus levels in toxic overdose,^8-12^ as they are both considered inducers of CYP3A4, but does it actually make a difference? We pose this question by presenting a case of tacrolimus toxicity in a patient with history of HSCT.

## Case Presentation

A 60-year-old man with a history of acute myeloblastic leukemia status postallogenic HSCT 2 months prior, tonic-clonic seizures, coronary artery disease, polycystic kidney disease, and hypertension presented with fever, diarrhea, and abdominal pain. He had been taking tacrolimus 0.5 mg BID, and levetiracetam 500 mg BID. He was transferred to the intensive care unit for persistent hypotension and acute hypoxic respiratory failure requiring intubation. Initial vitals included a temperature 100.2°F, blood pressure 70/43 mm Hg, heart rate 89 beats per minute, respiratory rate 20 breaths per minute, and 92% on room air. Pertinent physical examination findings included pallor and right upper quadrant abdominal tenderness with a positive Murphy’s sign. Initial laboratory tests were normal except for hemoglobin 10.1 g/dL, hematocrit 29.1%, platelets 79 000/µL (130 000-450 000/µL), sodium 133 mmol/L (136-144 mmol/L), bicarbonate 20 mmol/L (22-32 mmol/L), blood urea nitrogen 21 mg/dL, creatinine 2 mg/dL, and albumin 3.4 g/dL (3.5-5 g/dL). Initial arterial blood gas showed pH 7.37, pCO_2_ 30.9 mm Hg; pO_2_ 66 mm Hg; and a base excess of −8. Blood cultures were positive for *Pseudomonas aeruginosa*.

He was diagnosed with septic shock and started on acyclovir, voricanazole, vancomycin, tobramycin, metronidazole, and cefepime for empiric coverage given his immunocompromised state. Later that day, vasopressors (norepinephrine, epinephrine, vasopressin, and phenylephrine) were initiated along with stress dose steroids (hydrocortisone 100 mg TID). Empiric antibiotics were adjusted to meropenem instead of flagyl and cefepime. That night, the patient became progressively acidotic (pH of 7.37 earlier to pH of 7.22); therefore, it was decided to initiate continuous renal replacement therapy.

**Figure 1.**
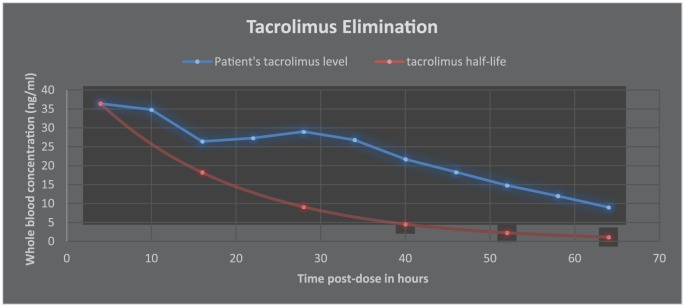
Whole blood tacrolimus concentration.

**Figure 2.**
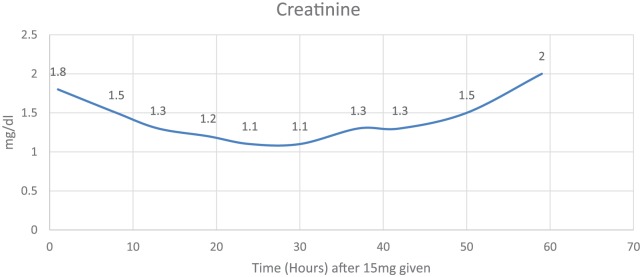
Creatinine levels.

The following day his tacrolimus level was 8.6 ng/mL (10-20 ng/mL) and creatinine was 2.2 (baseline = 1.8). The patient inadvertently received 15 mg intravenous tacrolimus instead of his scheduled 0.5 mg intravenous. Four hours later, a random tacrolimus level was 36.4 ng/mL. Tacrolimus was subsequently discontinued. Based on several case studies and literature review, phenytoin 200 mg BID was started for 4 doses and rifampin was started for 2 doses at 600 mg in order to potentially limit the patient’s tacrolimus toxicity. Levetiracetam was stopped during the administration of phenytoin; and rifampin was discontinued after 2 doses given its side effect profile. Sixteen hours postinjection, the tacrolimus level decreased to 26.4 ng/mL and to 9 ng/mL after 64 hours (Figure 1). Creatinine decreased to 1.1 after 30 hours (Figure 2). He was extubated 5 days later without any new neurological findings and his creatinine returned to baseline.

## Discussion

Our patient received 30 times his daily dose resulting in high tacrolimus levels. Tacrolimus is metabolized by hepatic CYP3A4 enzyme to inactive metabolites.^13,14^ In addition to the metabolism of tacrolimus by the CYP3A4 and CYP3A5 enzymes, an efflux transporter called P-glycoprotein plays a major role in the absorption of tacrolimus.^15^ As tacrolimus clearance is predominately through the biliary tract into the feces, there is a less than 1% of tacrolimus metabolites located in the urine.^10,16^ The elimination half-life of tacrolimus is approximately 12 hours (with a range of 3.5-40.5 hours).^17^ This half-life could potentially be decreased using CYP3A4 inducers. Phenytoin and rifampin^18^ are potent inducers of CYP3A4, theoretically increasing metabolism of tacrolimus into inactive metabolites. This therapy for tacrolimus toxicity has been successful based on previous case reports.^8-12^

Assuming there was sufficient time for distribution and to reach a steady state (at least 4 half-lives), our patient’s half-life increased to 37 hours compared with the reported half-life of 12 hours.

There are some possibilities for this increase in tacrolimus half-life, which can include ineffective or harmful effects of the phenytoin/rifampin combination. Given the fact that phenytoin and rifampin influence the same enzymes, CYP3A4, there is the chance of competition to the receptor, thereby removing effective binding of the medications to induce the enzyme. This is also supported by the fact that the tacrolimus levels dropped even quicker once the rifampin was discontinued at 24 hours (Figure 1).

**Figure 3.**
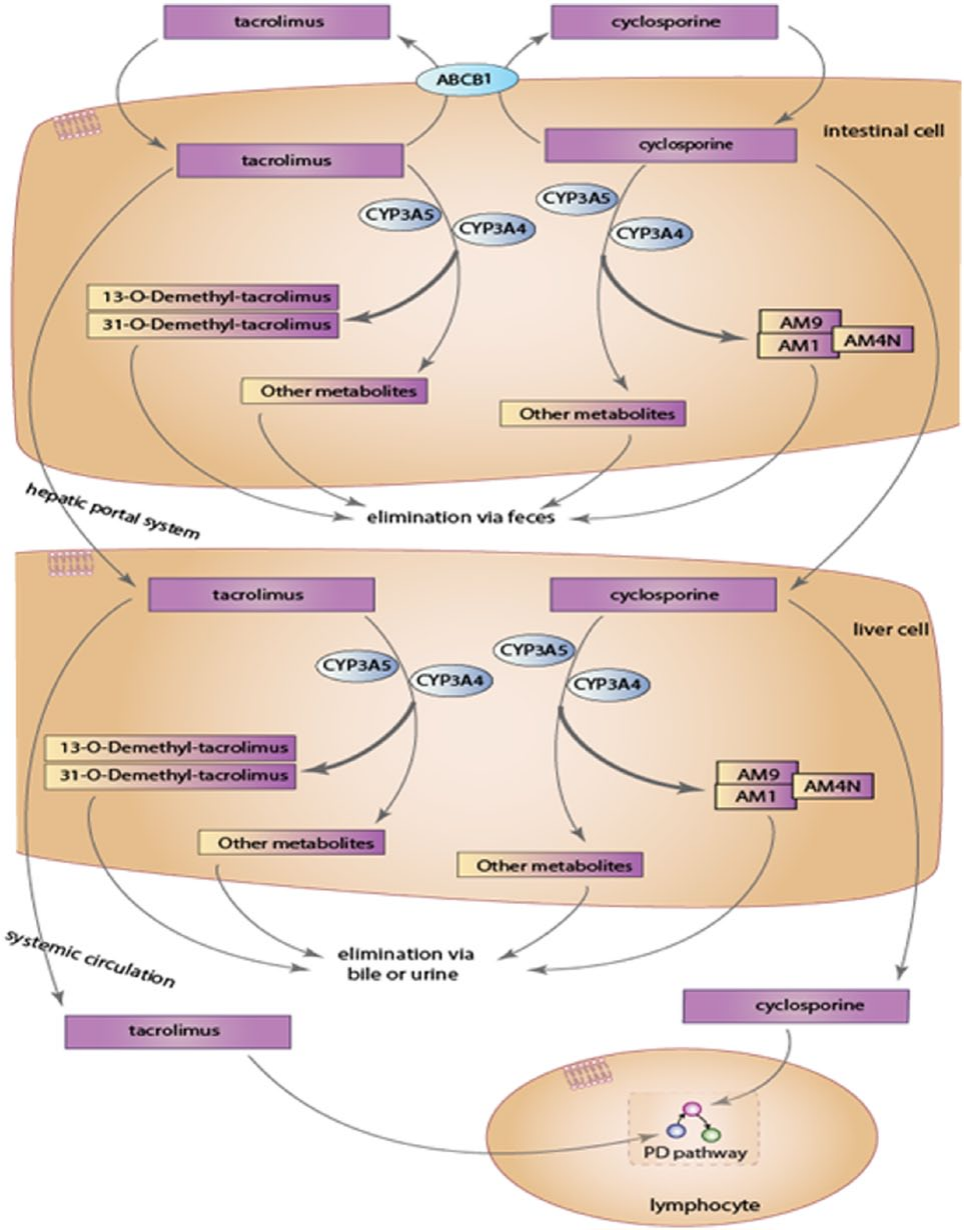
Metabolism of tacrolimus.^19^

Another explanation of our results is the change in metabolism kinetics at high concentrations. The likelihood of the eliminating mechanisms of tacrolimus (Figure 3) was already saturated, thereby preventing physiologic metabolism of the drug. Secondary to the toxic level of tacrolimus and theory of oversaturation, there could be insufficient time for P-glycoprotein transporter to function.

One must take into consideration the impact of voriconazole in this case. Studies have shown that concomitant use of tacrolimus and voriconazole, both competitive inhibitors of the CYP3A4 isoenzyme, requires tacrolimus dose reduction.^20^ Voriconazole is a competitive inhibitor for the cytochrome P450 3A4, 2C9, and 2C19 isoenzyme systems.^21,22^ Prior studies show measurement of baseline tacrolimus concentrations, followed by serial drug blood concentrations after the addition of voriconazole resulted in median tacrolimus blood concentrations increase of 115% above the target range.^21^ Even though voriconazole was initiated the day prior to the tacrolimus overdose and discontinued the following day, this still could allow sufficient time to affect the elimination of tacrolimus.

Rifampin and phenytoin influence the pharmacokinetics of voriconazole and may influence tacrolimus metabolism. Geist et al^23^ concluded that coadministration of voriconazole and rifampin will result in loss of therapeutic efficacy of voriconazole due to the massive reduction of systemic voriconazole exposure due to induced metabolism. Purkins et al^24^ concluded that it is necessary to double the dose of voriconazole in order to maintain its therapeutic plasma concentrations in the setting of phenytoin. This suggests that phenytoin and rifampin decrease the plasma concentrations of voriconazole, thus counteracting the CYP3A4 inhibitory effects to have little influence on tacrolimus metabolism.

Another consideration to help understand the increase in half-life could be related to patient-specific factors. For instance, the patient was critically ill resulting in the possibility of his physiological systems not operating at baseline. Furthermore, his genetics could lack sufficient physiologic function of the CYP3A4 enzyme. This possibility was eliminated after the calculation of his baseline tacrolimus half-life of 14.1 hours. This was calculated using tacrolimus concentrations a month prior to the incident when the patient was not critically ill and without phenytoin and rifampin on board. This could also be researched further by inspecting our patient’s individualized pharmacogenetics.

Age can also be considered another patient-specific factor that may affect the pharmacokinetics of tacrolimus. Pediatric transplant recipients require 2- to 4-fold higher doses of tacrolimus than adults to maintain similar trough concentrations.^25-32^ This could be related to differences in bowel length, hepatic blood flow, and P-glycoprotein expression,^33^ and there also appears to be maturation differences in expression of CYP3A4 and 3A5.^34^ For example, CYP3A5 is expressed in nearly 50% of all infant livers, but could be found in only 29% of adult livers.^34^ Based on the variability of age-related tacrolimus pharmacokinetics, our patient may have had limited functioning of his CYP3A4 enzymes due to his age of 60 years, thereby slowing the elimination of such drug. With respect to age-related absorptive properties of tacrolimus, it is currently unknown whether P-glycoprotein expression changes with age.^35^ Existing case reports of tacrolimus toxicity have been seen with patients from 9 years up to 70 years of age^8-12^; therefore, our patient fits into this age profile.

## Conclusion

There are numerous possibilities to explain why the half-life of tacrolimus increased despite adding phenytoin and rifampin. Further studies are recommended to ensure that phenytoin and rifampin are safe and effective treatment options to reduce levels in tacrolimus toxicity.
